# Anti-Hyperlipidemia, Hypoglycemic, and Hepatoprotective Impacts of Pearl Millet (*Pennisetum glaucum* L.) Grains and Their Ethanol Extract on Rats Fed a High-Fat Diet

**DOI:** 10.3390/nu14091791

**Published:** 2022-04-25

**Authors:** Nadiah S. Alzahrani, Ghedeir M. Alshammari, Afaf El-Ansary, Abu ElGasim A. Yagoub, Musarat Amina, Ali Saleh, Mohammed Abdo Yahya

**Affiliations:** 1 Department of Food Science and Nutrition, College of Food Science and Agriculture, King Saud University, Riyadh 11451, Saudi Arabia; 439203393@student.ksu.edu.sa (N.S.A.); amohammed4@ksu.edu.sa (A.E.A.Y.); 442106909@student.ksu.edu.sa (A.S.); mabdo@ksu.edu.sa (M.A.Y.); 2 Central Research Laboratory, Female Campus, King Saud University, Riyadh 11472, Saudi Arabia; elansary@ksu.edu.sa; 3 Department of Pharmacognosy, Pharmacy College, King Saud University, Riyadh 11451, Saudi Arabia; mamina@ksu.edu.sa

**Keywords:** *Pennisetum glaucum* grains, ethanol extract, hyperlipidemia, hypoglycemia, hepatoprotection, inflammation

## Abstract

This study tested the anti-hyperlipidemic, hypoglycemic, hepatoprotective, and anti-inflammatory effects of whole pearl millet grain powder (MPG) and its ethanol extract (MPGethaolE) in obese rats fed a high-fat diet. The rats were divided into eight groups based on the treatments they received: control, high fat diet (HFD), HFD + MGE (25 mg/Kg), HFD + MPGethaolE (50 mg/Kg), HFD + MPGethaolE (100 mg/Kg), HFD + MPG (10%), HFD + MPG (20%), and HFD + MPG (30%). The final body weight, visceral, epididymal fat pads, and the liver weight were significantly decreased, in a dose-dependent manner, in HFD fed rats that were co-administered either the MPG powder or MPGethaolE. In the same line, serum levels of triglycerides (TGs), cholesterol (CHOL), and low-density lipoprotein-cholesterol (LDL-c), as well as fasting glucose, insulin, HOMA-IR, and serum levels of lipopolysaccharides (LPS), interleukine-6 (IL-6), interleukine-10 (IL-10), C-reactive protein (CRP), tumor necrosis factor (TNF-α), and adiponectin were progressively decreased while serum levels of high-density lipoproteins (HDL-c) were significantly increased when increasing the doses of both treatments. In conclusion, both the raw powder and ethanolic extract of MP have a comparative dose-dependent anti-obesity, hypoglycemic, hypolipidemic, anti-inflammatory, and anti-steatotic in HFD-fed rats.

## 1. Introduction

Obesity is a rapidly increasing global problem that affects both sexes of all ages, which results from increased calories intake (westernization) and reduced energy expenditure [1]. Obesity is associated with socioeconomic problems and is a major leading cause of the development of main components of metabolic syndrome (Mets), including insulin resistance (IR), hypertension, atherogenic hyperlipidemia, and hyperglycemia [2,3]. In addition, these connected disorders are true risk factors for the development of cancer and cerebrovascular disorders, including coronary heart disease (CHD) and stroke [3].

Excess weight gain causes an increased risk for several diseases, most notably cardiovascular diseases, diabetes, and cancers [4]. Currently, weight loss by exercise or dietary intervention is the best-described strategy to alleviate obesity and associated comorbidities [5].

In addition, obese and metabolically unhealthy individuals have a state of low-grade inflammation that stimulates hepatic de novo lipogenesis, oxidative stress, and inflammation which results in the induction of non-alcoholic fatty liver disease (NAFLD), a condition that is characterized by high liver fat content [6,7]. The pathogenesis of NAFLD is complicated and is believed to result from increased hepatic oxidative stress and inflammation [8,9,10,11]. Indeed, higher levels of circulatory and hepatic markers of oxidative stress and inflammation are seen in obese metabolically active patients who developed NAFLD [12]. Within this view, once peripheral IR is established in obesity, the increasing influx of free fatty acids and inflammatory cytokines, and adipokines (i.e.**,** adiponectin) from the impaired adipose tissue to the liver results in a state of oxidative stress and inflammation due to the activation of Kupfer cells, mitochondria damage, endoplasmic reticulum (ER) stress, and increased production of reactive oxygen species (ROS) [9,13,14]. In addition, hepatic oxidative stress and inflammation are the leading mechanisms that stimulate hepatic lipotoxicity and gluconeogenesis by affecting several lipogenic transcription factors [i.e.**,** the sterol response element-binding protein (SREBPs)] and genes and inducing hepatic IR [9,15]. Furthermore, locally produced ROS and inflammatory cytokines stimulate the progression from simple steatosis to non-alcoholic steatohepatitis (NASH), as well as the development of liver fibrosis and hepatocellular carcinoma [14]. Therefore, it seems reasonable that drugs with hypoglycemic, hypolipidemic, antioxidant, and/or anti-inflammatory properties could afford protection against obesity-mediated NAFLD and liver damage [11,16,17,18].

Apart from traditional drugs, herbal foods are currently under focus for the treatment of obesity, Mets, T2DM, and NAFLD [19,20]. Grains such as oats, quinoa, and barley are reported to have anti-hyperglycemic, anti-hyperlipidemic, and anti-inflammatory effects on humans and rats [21,22,23,24]. Millet is a grain that is utilized as a traditional food for many populations in various countries worldwide, particularly in arid and semi-arid areas [25,26], including Saudi Arabia [26]. In addition, millet grains have higher antioxidants and potential than flaxseeds, quinoa, and chia seeds [25,27,28]. Nutritionally, millet contains more nutrients than rice or wheat [24]. Millet contains lysine, methionine, and threonine higher by 40%, 30%, and 20%, respectively, than corn [26]. It also contains a considerable number of insoluble fibers [23]. Millet is classified as a low-glycemic index (GI) food due to its high fiber content. So, it is characterized as a food with a low-glycemic index (GI). The GI assesses how much the carbohydrate content of food influences the rate and extent of change in post-prandial blood glucose concentration. The general dietary approach for managing glycemic control is to consume low-GI foods [29,30]. Reportedly, millets, as a low-GI food, help lower blood glucose available for triacylglycerol synthesis. Furthermore, millets reduced VLDL cholesterol, a carrier of triacylglycerol in plasma, lowering triacylglycerol levels even further. As a result, the consumption of millet grains may play an important role in lowering the level of blood lipids [30,31]. Many kinds of bioactive compounds, such as polyphenols, mostly flavonoids and phenolic acids, naturally occur in millet. They are secondary metabolites of plants that fight against environmental and microbial threats [32,33]. Polyphenols offer many health benefits, seen in their antioxidant and anti-inflammatory properties [34,35]. In light of the foregoing, this study aims to assess the effects of administering different concentrations of millet grain, as well as different doses of its methanol extract, on biomarkers linked to CVDs such as glucose, insulin, HOMA-IR, lipid profile, pro-inflammatory biomarkers, and anti-inflammatory biomarkers in rats fed a high-fat diet.

## 2. Materials and Methods

### 2.1. Animals

Forty-eight adult male Wister rats (150 ± 20 g, ten weeks old) were obtained and maintained in the Experimental Animal Care Center at King Saud University, Riyadh, Saudi Arabia. For one week of the acclimatization period, the rats were kept in controlled ambient conditions (22 ± 2 °C, 50% humidity, 12/12 h light/dark). Animals were allowed to access their diets and drinking water freely. The Research Ethics Committee at King Saud University, Riyadh, Saudi Arabia, approved the experimental protocol (Ref. No.: KSU-SE-21-49).

### 2.2. Diets

Both control (3.85 Kcal/g) (Cat. No. D12450H) and HFD (3.85 Kcal/g) (Cat. No. D12451) were purchased from Research Diets, New Brunswick, NJ, USA. The HFD was designed to induce obesity, IR, and NAFLD after feeding for eight concessive weeks. The energy obtained from fat, proteins, and carbohydrates for each diet is shown in Table 1. The ingredients of both diets can be obtained from the company site.

### 2.3. Preparation of the Raw Powder and Ethanoic Extract

The pearl millet (PM) (*Pennisetum glaucum* L.) grains were purchased from a local market in the Al Baha region, Saudi Arabia, and grounded into a fine powder with a commercial grinder (SANYO-Made in Japan). The flour MP whole grain (MPG) powder was stored in a dry, clean place for next use. To prepare the ethanolic extract from the PM grain powder (MPGethaolE), 2 Kg of the PM flour was suspended in 2.5 L of ethanol (70% purity) with continuous shaking for 3 h. This procedure was repeated three times. Then, the obtained extract was filtered using filter paper (Whatman No. 1). The extract was concentrated using a rotary vacuum (60 °C, 450 mmHg). The dried residues were placed in tightly closed glass tubes and stored at −20 °C for later use [36]. At the time of the experiments, MPG powder and MPGethanolE residues were dissolved in 0.1% DMSO as a carrier solvent.

### 2.4. Experimental Design

The rats were divided into eight groups (*n* = 6/group); a control group that was orally administered 0.25 mL of 0.1% DMSO (vehicle), and another seven HFD-fed rats that were treated as follows; one group only fed HDF and orally administered 0.25 mL of 0.1% DMSO; three groups fed HFD and concomitantly orally administered the MG powder solution (0.25 mL) at increasing concentrations of 10%, 20%, or 30%, respectively; and another three groups which were fed HFD and concomitantly orally administered MPGethaolE solution (0.25 mL) at increasing concentrations of 25; 50; or 100 mg/kg. All treatments were administered daily by gavage using a stainless-steel feeding cannula by a well-trained vet and continued for eight weeks. Throughout the experimental period, the rats’ body weight and food intake were recorded weekly.

### 2.5. Doses Selection

The doses of millet ethanol extract used in experiments were adapted from Luqman et al. [37], whereas whole-grain doses were adapted from Khan et al. [38].

### 2.6. Animal Anesthesia and Collection of Blood Samples and Tissues

All animals were fasted overnight and then anesthetized with an intraperitoneal dose of ketamine hydrochloride (90 mg/Kg), and xylazine hydrochloride (10 mg/Kg) [39]. Blood (1 mL) was directly collected by cardiac puncture into plain tubes. The tubes were then centrifuged (3000× *g*; 10 min) at room temperature to collect the serum. All serum samples were collected in new tubes and stored at −20 °C for further biochemical analysis. Then, all rats were exposed to euthanasia using the cervical dislocation protocol. The livers and intestines were collected on ice and cut into smaller pieces. Parts of the livers were placed in 10% buffered formalin. Other livers were snap-frozen in liquid nitrogen and stored at −80 °C for biochemical analysis.

### 2.7. Biochemical Analysis

Serum glucose and insulin levels were measured using rats’ colorimetric and ELISA kits (Cat No. 10009582 Cayman Chemical, MI, USA and Ca., No. KA3811, Abnova, CA, USA, respectively). The hemostasis model of insulin resistance was calculated as described by Salgado et al. [40], using the following formula: HOMA-IR fasting insulin (ng/mL) × fasting glucose (nmol/L)/22.5. Serum levels of CHOL and TGs were measured using rats’ specific assay kits (Cat. No. ECCH-100, BioAssay Systems, Cat. No. 10010303, Cayman Chemical, MI, USA. Serum levels of HDL-c and LDL-c were determined using a colorimetric kit (Cat. No. K613-100, BioVision, CA, USA). Serum levels of tumor Necrosis Factor-alpha (TNFα), Interleukin 6 (IL-6), and Interleukin 10 (IL-10) were measured using rats’ ELISA kits (Cat. No. MBS2507393; Cat. No. MBS175908, Cat. No. MBS824577, Shirley, NY, USA, respectively). ELISA test was used to determine C-reactive protein (CRP) and adiponectin levels in serum (Cat. No. ab256398; Cat. No. Ab239421, Abcam, Cambridge, UK). Serum levels of lipopolysaccharides (LPS) were measured using An ELISA kit. All protocols were conducted as instructed by each kit manufacturer for *n* = 6 rats/group. All standard curves generated by these kits are shown in the Supplementary File (S1).

### 2.8. Light Microscope

Fresh liver samples were fixed in buffer solution (10% formalin). The tissues were dehydrated in ethanol at increasing concentrations (70–100%) and cleared with Xylene. The tissues were then embedded in the paraffin wax, sliced into 4-μm thick sections, stained with hematoxylin and eosin, and examined by an independent investigator under a light microscope (Olympus Optical, Tokyo, Japan).

### 2.9. Statistical Analysis

The statistical analyses were accomplished using Graph Pad Prism analysis software (Version 8, San Diego, CA, USA). Comparisons between animal groups were carried out using one-way ANOVA followed by Tukey’s *t*-test. The alpha level for statistical significance determination was set at 0.05.

## 3. Results

### 3.1. Changes in Body Weight and Adiposity Markers

**Figure 1:**
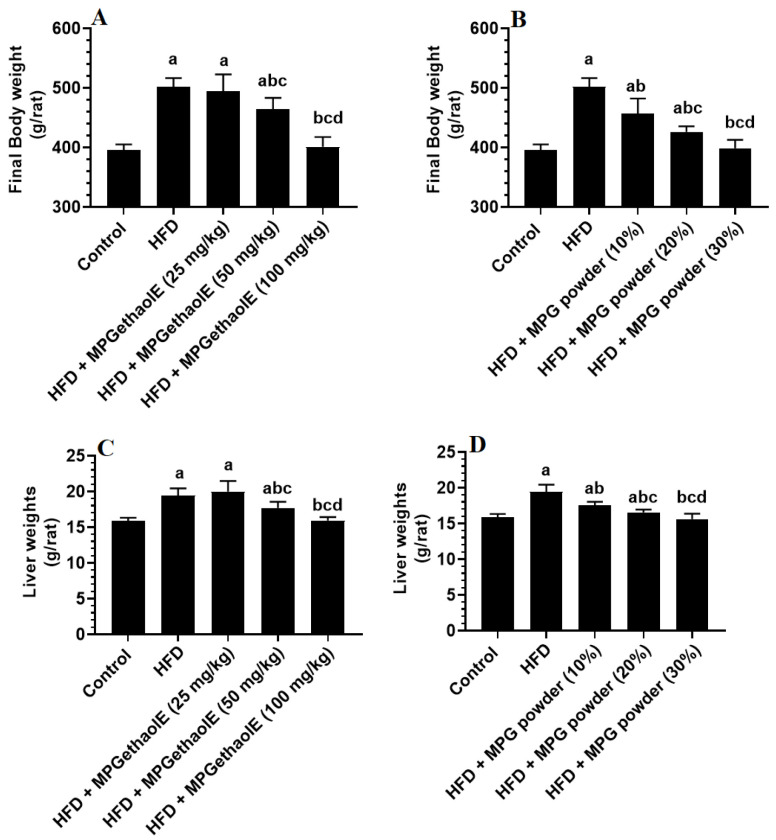
Final body (**A**,**B**) and liver weights (**C**,**D**) in all groups of rats. Data were analyzed by 1-way ANOVA followed by Tukey’s *t*-test as post hoc. Data were considered as mean + SD and considered as significantly different at *p* < 0.05. a: significantly different vs. the control rats. b: significantly different vs. HFD, c: significantly different vs. HFD + MPGethaolE (25 mg/kg) or MPG powder (10%), d: significantly different vs. HFD + HFD + MPGethaolE (50 mg/kg or MPG powder (20%).

**Figure 2:**
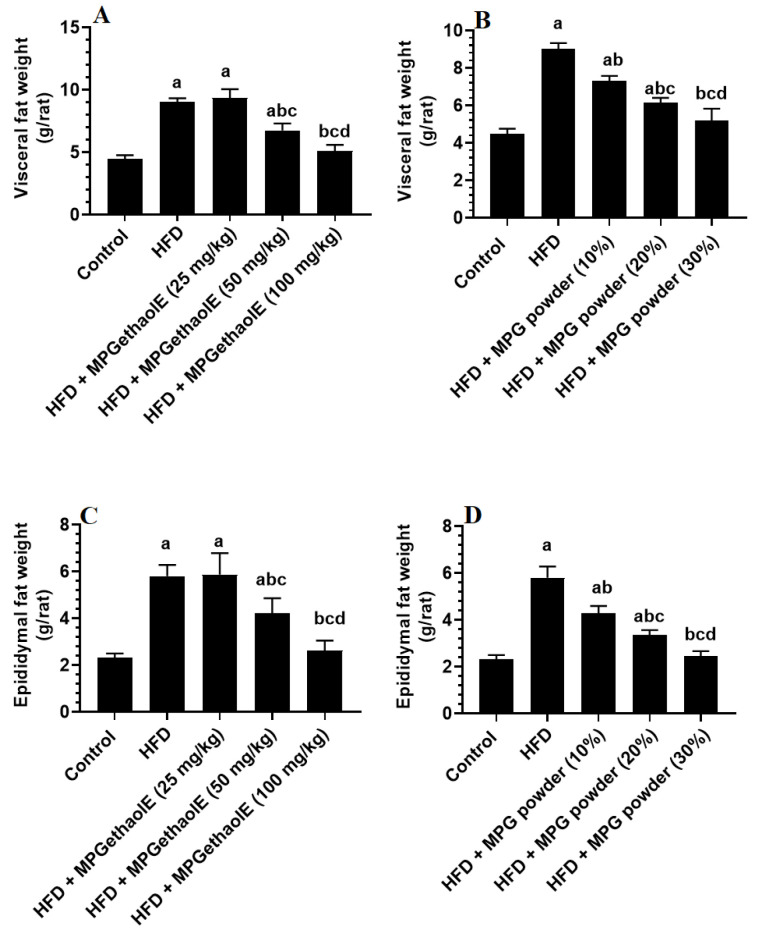
Weights of visceral (**A**,**B**) and epididymal (**C**,**D**) fat pads in all groups of rats. Data were analyzed by 1-way ANOVA followed by Tukey’s *t*-test as post hoc. Data were considered as mean + SD and considered as significantly different at *p* < 0.05. a: significantly different vs. the control rats. b: significantly different vs. HFD, c: significantly different vs. HFD + MPGethaolE (25 mg/kg) or MPG powder (10%), d: significantly different vs. HFD + HFD + MPGethaolE (50 mg/kg or MPG powder (20%).

**Figure 3:**
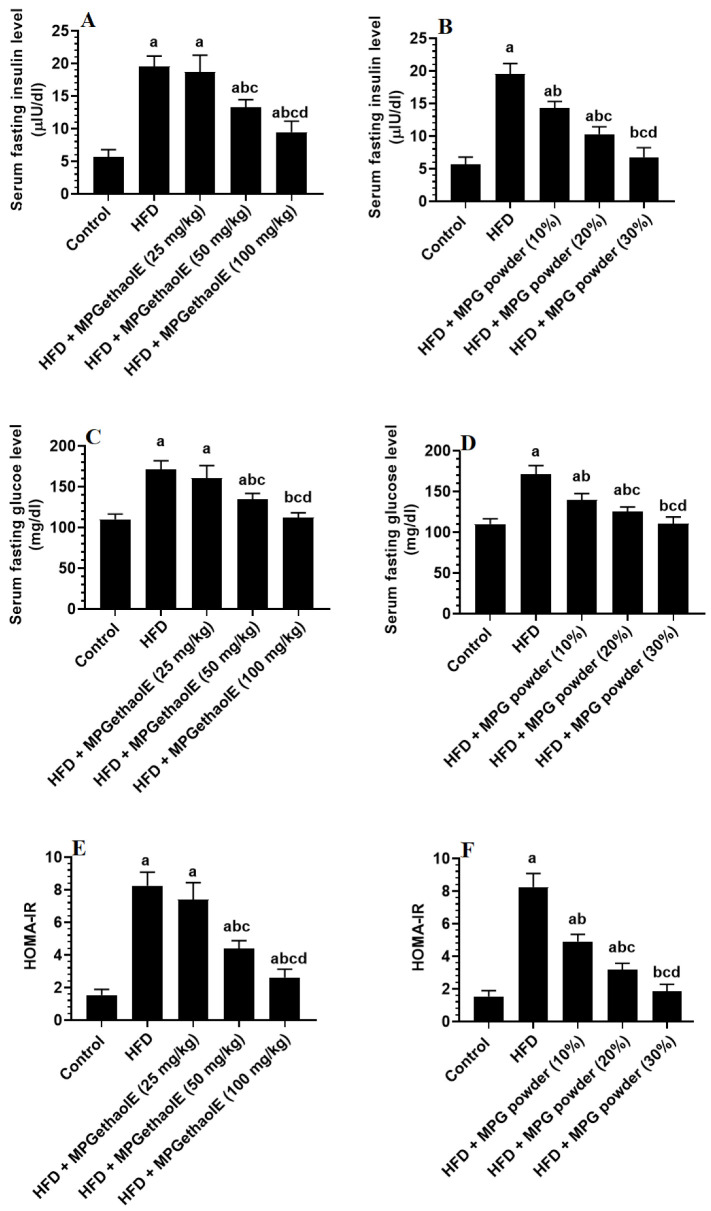
Fasting insulin (**A**,**B**) and glucose (**C**,**D**), as well as Homeostatic Model Assessment for Insulin Resistance (HOMA-IR) (**E**,**F**) levels in the plasma of all groups of rats. Data were analyzed by 1-way ANOVA followed by Tukey’s *t*-test as post hoc. Data were considered as mean + SD and considered as significantly different at *p* < 0.05. a: significantly different vs. the control rats. b: significantly different vs. HFD, c: significantly different vs. HFD + MPGethaolE (25 mg/kg) or MPG powder (10%), d: significantly different vs. HFD + HFD + MPGethaolE (50 mg/kg or MPG powder (20%).

Final body, liver, visceral fat, and epididymal fat pad weights, as well as fasting glucose and insulin levels and values of HOMA-IR were significantly increased in HFD-fed rats as compared to normal diet-fed rats (control) (Figure 1D, Figure 2A–D and Figure 3A–E). However, the levels of all of these markers were significantly reduced in HFD-fed rats after receiving either MPGethaolE (50 or 100 mg/Kg) or whole millet grain, MPG (10–30%) (Figure 1D, Figure 2A–D, and Figure 3A–E). In addition, this reduction in biomarkers was dose-dependent, with maximum effects being noticed with the extract dose of 100 mg/Kg and 30% MPG powder feeding.

### 3.2. Changes in Lipid Profile

**Figure 4:**
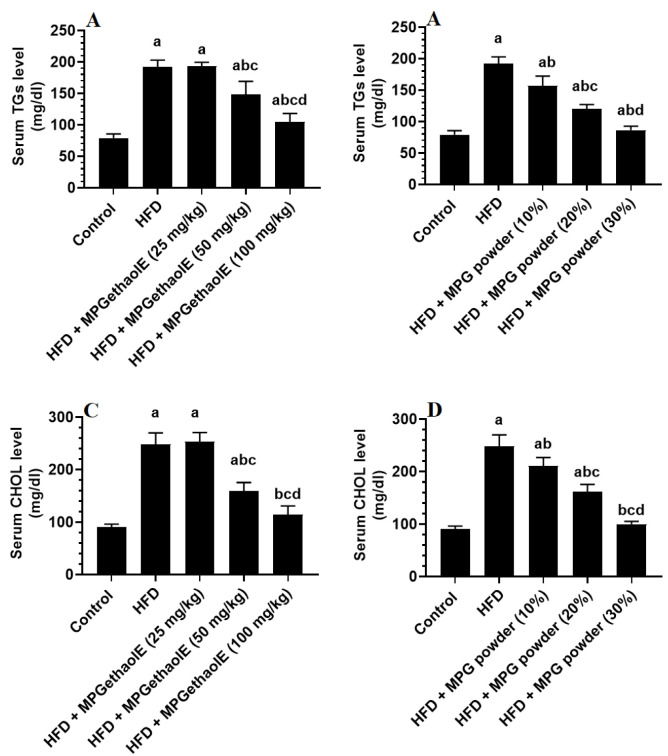
Serum levels of triglycerides (TGs) (**A**,**B**) and cholesterol (CHOL) (**C**,**D**) in all groups of rats. Data were analyzed by 1-way ANOVA followed by Tukey’s *t*-test as post hoc. Data were considered as mean + SD and considered as significantly different at *p* < 0.05. a: significantly different vs. the control rats. b: significantly different vs. HFD, c: significantly different vs. HFD + MPGethaolE (25 mg/kg) or MPG powder (10%), d: significantly different vs. HFD + HFD + MPGethaolE (50 mg/kg or MPG powder (20%).

**Figure 5:**
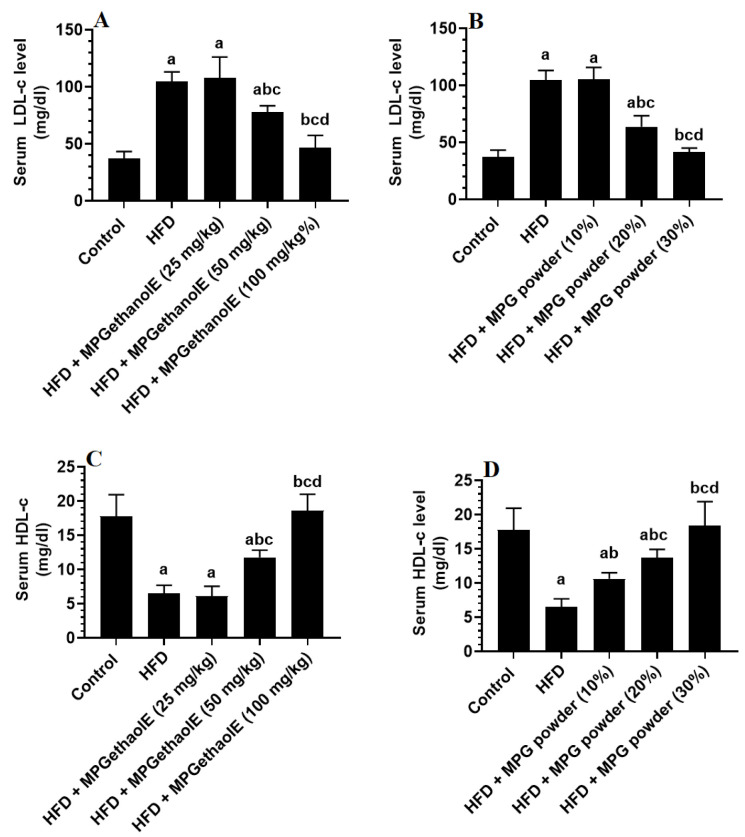
Serum levels of low-density lipoproteins (LDL-c) (**A**,**B**) and high-density lipoproteins (HDL-c) (**C**,**D**) in all groups of rats. Data were analyzed by 1-way ANOVA followed by Tukey’s *t*-test as post hoc. Data were considered as mean + SD and considered as significantly different at *p* < 0.05. a: significantly different vs. the control rats. b: significantly different vs. HFD, c: significantly different vs. HFD + MPGethaolE (25 mg/kg) or MPG powder (10%), d: significantly different vs. HFD + HFD + MPGethaolE (50 mg/kg or MPG powder (20%).

Serum levels of TGs, CHOL, and LDL-c were significantly increased, whereas serum levels of HDL were significantly decreased in HFD-fed rats as compared to control rats (Figure 4A–D and Figure 5A–D). The levels of these lipids in HFD + MGE extract treated rats (25 mg/kg) did not significantly differ from those in HFD rats. On the other hand, treating HFD rats with the MPGethaolE at doses of 50 or 100 mg/kg or with MPG powder at 10, 20, and 30% significantly reduced serum levels of TGs, CHOL, and LDL-c and increased serum levels of HDL-c as compared to HFD-fed rats (Figure 4A–D and Figure 5A–D). Of note, these effects were dose-dependent.

### 3.3. Changes in Inflammatory Mediators

**Figure 6:**
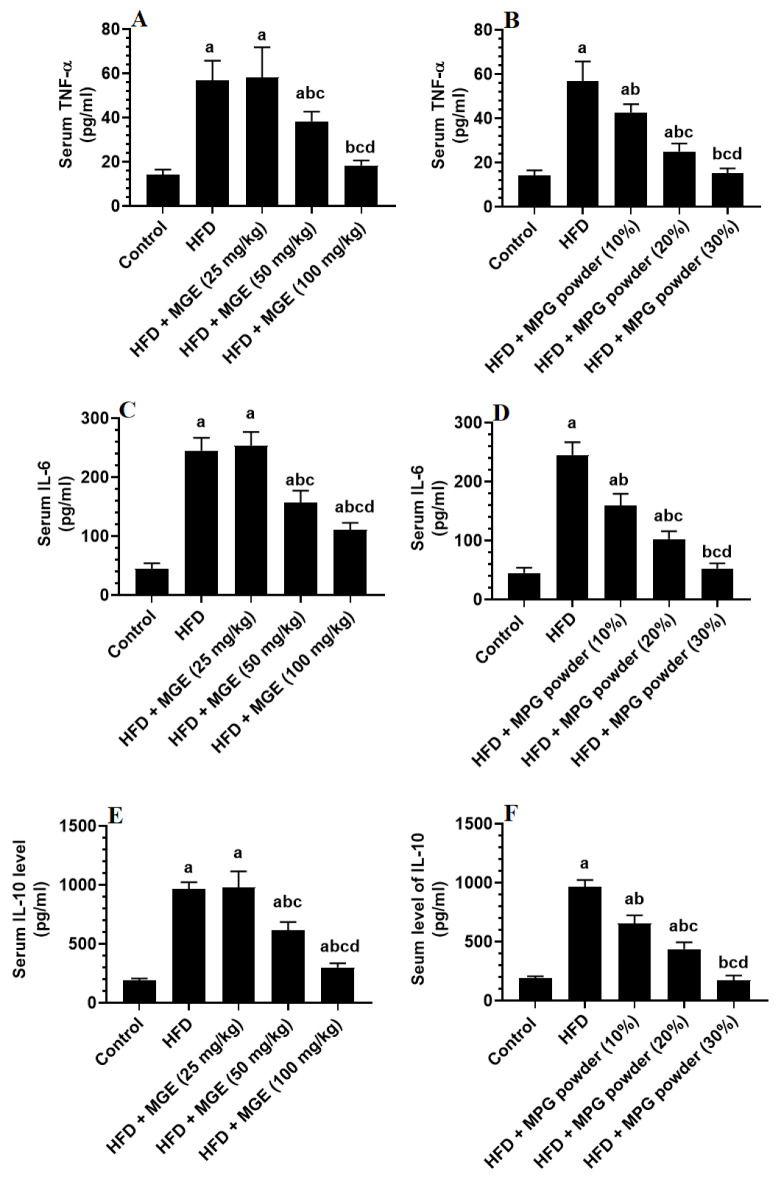
Levels of tumor necrosis factor-α (TNF-α) (**A**,**B**), interleukin-6 (IL-6) (**C**,**D**), and interleukin-10 (**E**,**F**) in the serum of all groups of rats. Data were analyzed by 1-way ANOVA followed by Tukey’s *t*-test as post hoc. Data were considered as mean + SD and considered as significantly different at *p* < 0.05. a: significantly different vs. the control rats. b: significantly different vs. HFD, c: significantly different vs. HFD + MPGethaolE (25 mg/kg) or MPG powder (10%), d: significantly different vs. HFD + HFD + MPGethaolE (50 mg/kg or MPG powder (20%).

**Figure 7:**
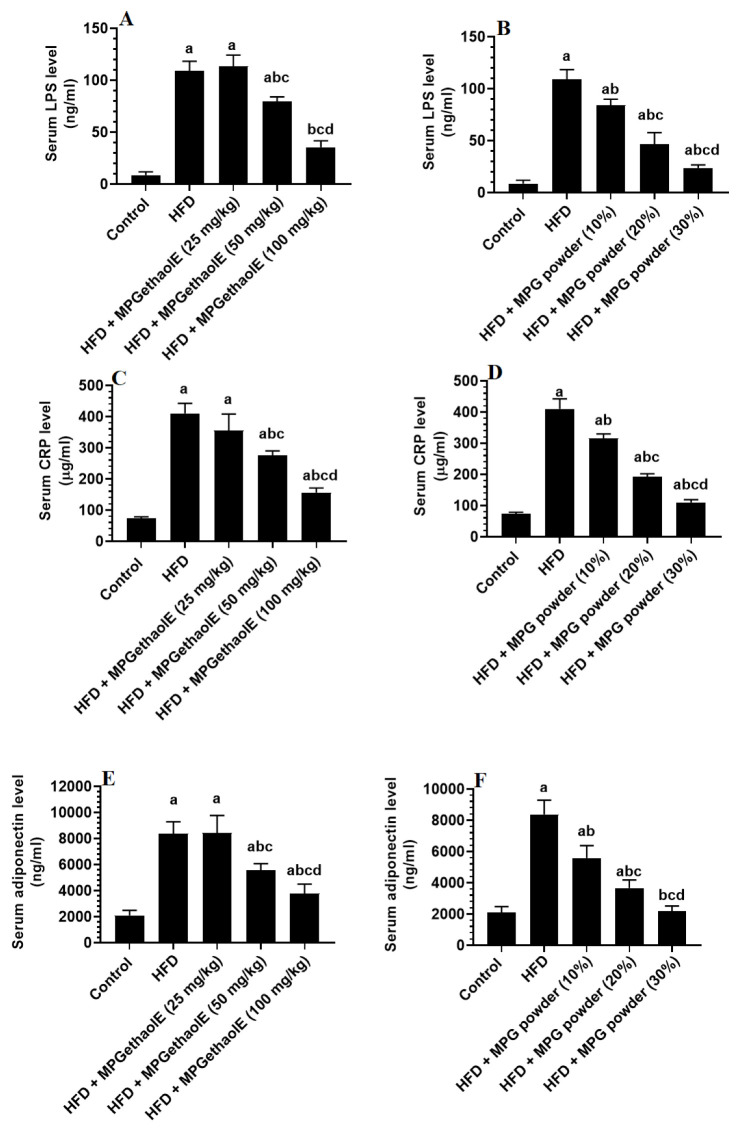
Levels of lipopolysaccharides (LPS) (**A**,**B**), C-reactive protein (**C**,**D**), and adiponectin (**E**,**F**) in the serum of all groups of rats. Data were analyzed by 1-way ANOVA followed by Tukey’s *t*-test as post hoc. Data were considered as mean + SD and considered as significantly different at *p* < 0.05. a: significantly different vs. the control rats. b: significantly different vs. HFD, c: significantly different vs. HFD + MPGethaolE (25 mg/kg) or MPG powder (10%), d: significantly different vs. HFD + HFD + MPGethaolE (50 mg/kg or MPG powder (20%).

TNF-α, IL-6, IL-10, CRP, and adiponectin levels in the blood serum were significantly higher in HFD-fed rats than in control rats but were statistically similar when compared with HFD rats administered the MPGethaolE (25 mg/Kg) (Figure 6A–F and Figure 7A–F). In comparison to HFD-fed rats, serum levels of all inflammatory markers were significantly reduced in rats administered HFD + MPGethaolE (50 and 100 mg/Kg), as well as whole MPG powder (10, 20, and 30%), in a dose-dependent manner (Figure 6A–F and Figure 7A–F).

### 3.4. Improvement in Liver Histology

**Figure 8:**
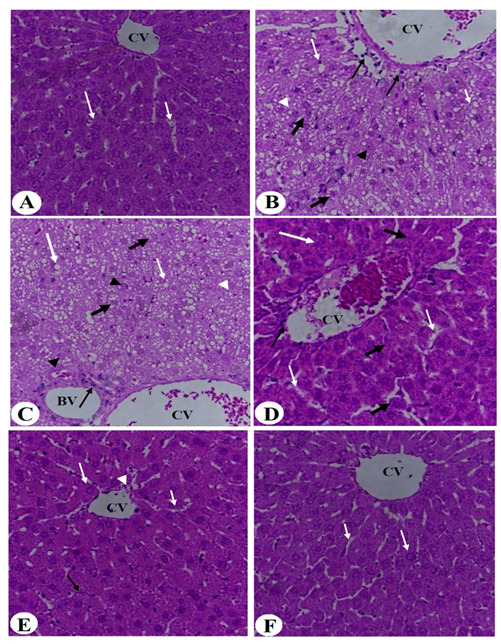
Histological images of the liver of all groups of rats. (**A**) was taken from a control rat and showed intact hepatocytes (long white arrow) radiating from the central vein (CV) with intact sinusoids (short white arrow). (**B**,**C**) were taken from HFD-fed and HFD + MPGethaolE (25 mg/kg) and showed severe hepatic fat accumulation of large (long white arrow), medium (short white arrow), and small (white arrowhead) fat vacuoles. The hepatocytes showed increased karyolysis (thick black arrow) and Pyknotic (black arrowhead) nuclei. Besides, increased inflammatory cells were seen around the dilated CV and the blood vessels (BV) (long thin black arrow). (**D**) was taken from HFD + MPGethaolE (50 mg/kg) and showed much improvement in the structure of the hepatocytes with almost no fat accumulation and improvements in the structure of the hepatocytes (long white arrow). However, some hepatocytes showed swelling and abnormal appearance (Thick black arrow) and dilated sinusoids (short white arrow) with increased inflammatory cells around the damaged CV (thin black arrow). (**E**,**F**) were taken from HFD + MPGethaolE (50 mg/kg) and show almost normal hepatic structure with intact hepatocytes (long white arrow) and normally sized CV and sinusoids (short white arrow). Very few hepatocytes showed damage.

**Figure 9:**
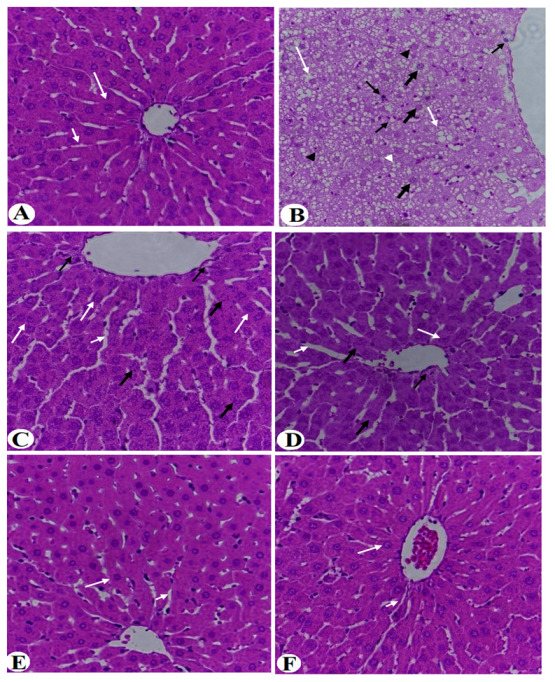
Histological images of the liver of all groups of rats. (**A**) was taken from a control rat and showed intact hepatocytes (long white arrow) radiating from the central vein (CV) with intact sinusoids (short white arrow). (**B**) was taken from an HFD-fed rat and showed severe hepatic fat accumulation of large (long white arrow), medium (short white arrow), and small (white arrowhead) fat vacuoles. The hepatocytes showed increased karyolysis (thick black arrow) and Pyknotic (black arrowhead) nuclei. Besides, increased inflammatory cells were seen around the dilated CV and the between cells (long thin black arrow). (**C**,**D**) was taken from HFD + MPG powder (10%) and showed much improvement in the structure of the hepatocytes with almost no fat accumulation and normally appeared hepatocytes (long white arrow). However, some hepatocytes showed swelling and abnormal appearance (Thick black arrow) and dilated sinusoids (short white arrow). (**E**,**F**) were taken from HFD + MPGethaolE (100 mg/kg) or MPG powder (30%, respectively) and show almost normal hepatic structure with intact hepatocytes (long white arrow) and normally sized CV and sinusoids (short white arrow).

Liver taken from the control rats showed regular hepatic features with intact hepatocytes radiating from an intact central vein (Figure 8A and Figure 9A). Livers obtained from HFD-fed rats showed a massive increase in the number of fat vacuoles of all sizes (i.e., large, medium, and small) where most of the hepatocytes showed karyolysis and Pyknosis (Figure 8B and Figure 9B). In addition, these livers showed abnormally dilated CV and blood vessels (BV) and an increased number of invading inflammatory cells (Figure 8B and Figure 9B). Similar pathological changes to those seen in the HFD-fed rats were also seen in HFD + MPGethaolE (25 mg/kg) (Figure 8C). On the other hand, much improvement in the structure of the hepatocytes and a reduction in fat vacuolization, inflammatory cell invasion, and central vein dilation were observed in the groups of HFD fed rats that received MPGethaolE doses of 50 and 100 mg/kg or MPG powder of 10, 20, and 30% (Figure 8D–F and Figure 9D–F). However, almost normal liver structure morphology was observed in rats administered HFD + MPGethaolE (100 mg/Kg) or HFD + MPG powder (30%) (Figure 8E,F and Figure 9E,F).

## 4. Discussion

In this study, we demonstrate that increasing doses of both the powder or ethanolic extract of MP can alleviate HFD-induced obesity, IR, and hepatic in rats in a dose-dependent manner. Additionally, we show that these beneficial effects are mediated, at least, by hypoglycemic, hypolipidemic, and anti-inflammatory potentials.

Chronic feeding of HFD is a common strategy to induce obesity and MetS in rats but not mice [41]. Obesity is the major triggering event that promotes the development of all other comorbidities, including IR, hypertension, hyperglycemia, hyperinsulinemia, and NAFLD, which could affect the overall health and promote liver damage [7]. In this study, the significant increase in body weights and visceral/epididymal fat pads weights demonstrates the role of HFD in promoting obesity in rats and support many other previous authors [42,43]. On the other hand, administration of both the powder and ethanolic extract of the MP grains attenuated the increase in final body and fat weights in HFD-fed rats in a dose-dependent manner, thus suggesting having a potent anti-obesity effect. These data support the findings of Murtaza et al. [44] who emphasized the importance of eating whole grains containing bran in reducing weight gain. In the same manner, administration of ethanol extract of seabuckthorn for 13 weeks prevented body weight gain [45]. This inhibitory effect of MP grains on body and fat weight fain could be explained by their high content of fibers and subsequent reduction in intestinal fat absorption [46,47]. In addition, whole millet grains can reduce body weights by decreasing adipose tissue mass by inhibiting adipocyte proliferation, increasing fat cell death, and impairing triglyceride absorption by inhibiting pancreatic lipase production [45,48]. However, our data still contradict those reported by Li et al. [49] who have shown no effect of millet grains’ doses of 10% and 50% on body weight and attributed that to millet’s high starch content. Such variation could be explained by the variation in millet species and treatment dose and period, as well as the variation in cultivation season and source of the plant.

Nonetheless, fasting hyperglycemia and hyperinsulinemia are major features of T2DM and are the major hallmarks associated with obesity and chronic HFD feeding [50]. Peripheral and hepatic IR is believed to be the major cause of such hyperglycemic conditions [50]. In addition, hepatic oxidative stress, inflammation, lipotoxicity, and lipid peroxidation are major triggers for hepatic IR, which stimulates glucose output and impairs insulin signaling [9,11]. This was also evidenced in the HFD-fed rats of this study, where these rats showed higher fasting levels of glucose and insulin, as well as higher levels of HOMA-IR. However, the significant reduction in the levels of fasting glucose and insulin levels, as well as values of HOMA-IR after the whole grain powder or ethanolic extract administration of MP is the strongest evidence for the hypoglycemic effect of this plant. Although fewer studies have examined the hypoglycemic effect of MP in experimental or clinical studies, some previous authors have shown potent hypoglycemic effects of different millet grain species, an effect that has been suggested to their role in decreasing lipid and glucose intestinal absorption [51,52]. Also, the administration of cooked millet significantly improved the glucose hemostasis throughout the activation of the PPARs signaling pathway, which may slow glucose production and promote energy balance, then alleviate glucose homeostasis disorders in diabetic mice [48,51]. On the other hand, some other authors have suggested that the hypoglycemic effect of millet is a direct effect mediated by its active proteins, especially prolamin, which can reduce the incidence of T2DM by attenuating hyperlipidemia and improving IR through inhibiting pancreatic lipase, suppressing lipoxygenase, and reducing TGs hepatic accumulation [51,53]. Propionate, another component found in millet, can also stimulate the release of glucagon-like peptide-1 (GLP-1) from enteroendocrine L cells to increase insulin’s responsiveness to glucose [49,54,55,56]. Also, in vitro studies have shown that ethanolic extracts from different types of millet can inhibit the activity of some enzymes involved in glucose metabolisms, such as α-glucosidase and α-amylase [57]. Moreover, millet grain can improve hepatic glucose hemostasis by alleviating obesity/T2DM-associated oxidative stress by its high content of antioxidant-derived ingredients [58].

On the other hand, obesity and increased adipose tissue mass are associated with increased inflammatory cytokines and adipokines release. Indeed, obese subjects and animal models have a state of low-grade inflammation and showed higher circulatory levels of different inflammatory cytokines such as TNF-α; IL-6, and CRP [59,60,61]. Also, obesity is associated with impaired adiponectin release from the adipocytes. Adiponectin is a potent antioxidant and anti-inflammatory hormone that can attenuate oxidative stress and suppress inflammation by decreasing ROS generation, inhibiting NF-𝜅B (a master inflammatory transcription factor), downregulating TNF-α, and stimulating the anti-inflammatory cytokine, IL-10. In general, obesity is associated with reduced serum adiponectin levels which increase with weight loss. However, 20% of obese individuals are metabolically healthy and have increased adiponectin levels, which could be unusual due to adiponectin resistance [62,63]. In the same line with these observations, our HFD-fed- rats also showed higher serum levels of adiponectin, TNF-α; IL-6, and CRP, which confirm the low-grade inflammatory state in these rats. However, a dose-response reduction in the levels of these inflammatory with a concomitant increase in the levels of IL-10 was observed in the serum of HFD-fed rats co-treated with MP powder or ethanol extract.

Although these adiponectin inhibitory and anti-inflammatory effects of MP could be secondary to the observed reduction in adipose tissue fat mass, other studies support those millets have independent anti-inflammatory effects mediated by targeting the synthesis of the inflammatory cytokines. Indeed, the administration of germinated millet flour prevented hepatic inflammation and steatosis in HFD-fed animals by decreasing the hepatic levels of TNF-α and stimulating those of IL-6 [64]. Also, the anti-inflammatory potential of the millet bran derived-bound polyphenols against LPS-induced inflammation in HT-29 cells was mediated by suppressing ROS and the activation of NF-κB, which subsequently downregulates IL-6 and TNF-α [65]. Millet-derived peptides also prevented LPS-induced inflammation in vivo and in vitro by suppressing TNF-α and IL-6 [66]. However, IL-10 plays a crucial role in regulating obese subjects, promoting weight loss, increasing insulin sensitivity, working as an int-inflammatory agent, and protecting from lung injury [67]. The increase in the levels of IL-10 in the serum of HFD could be a protective compensatory mechanism to reduce inflammation. This also supports the finding of Calcaterra et al. [68] who have shown higher levels of IL-10 in the serum of obese subjects, especially those with visceral fat deposition. In addition, the increase in serum levels of IL-10 could be a result of increasing adiponectin levels which is the best-known inducer of IL-10 [64,65,66,67,68]. If this is correct, then the reduction in the levels of this anti-inflammatory marker after MP powder or ethanol extract could be explained by the reduction in the levels of adiponectin, TNF-α, and IL-6.

Another important observation in this study is the ability of both the powder and ethanolic extract of MP to attenuate hepatic steatosis and associated hyperlipidemia in the HFD-treated rats. In obesity, the high peripheral influx of FFAs and inflammatory cytokines from the adipose tissue in response to IR exaggerates ROS and inflammatory cytokines production in the liver and promotes hepatic lipotoxicity and IR [9,13,14,69,70,71,72,73,74,75]. This is associated with sustained expression of lipogenic transcription factors, the sterol response element-binding protein 1c/2 (SREBP-1c and SREBP2), which stimulates TGs and CHOL synthesis, as well as reduced expression of the Peroxisome proliferator-activated receptor (PPAR)-alpha (PPARα) which stimulates FAs mitochondria oxidation [76,77,78,79]. The role of HFD in mediating hepatic steatosis and hyperlipidemia was also evidenced in HFD-fed rats of this study as demonstrated the higher circulatory levels of Serum TGs, CHOL, and LDL-c that were coincided with a significant reduction in the levels of HDL-c. In addition, the livers of these rats had higher weights and accumulated large fat vacuoles with ballooning, indicating progression to NASH. These data are in the same line as other studies which utilized the same animal model [54,80,81,82,83]. On the other hand, both MP powder and ethanol extract showed a comparative dose-dependent hypolipidemic effect in HFD-fed rats, which could be attributed to a direct effect or secondary to its anti-obesity (i.e., improving peripheral insulin sensitivity), hypoglycemic, antioxidant, and anti-inflammatory effects.

Consistent with our results, treatment with whole-grain highland hull-less barley reduced the fat accumulation in rats fed HFD due to their rich dietary fibers [84]. In this context, whole millet grain can reduce hepatic steatosis by decreasing TGs and CHOL intestinal absorption [46,47]. On the other hand, several plant-derived bioactive compounds may directly prevent NAFLD and hepatic steatosis by decreasing *de novo* lipogenesis through direct downregulating of the sterol regulatory element-binding protein 1c (SREBP-1c), increasing FAs oxidation through up-regulating the PPAR receptor and improving insulin sensitivity, and decreasing intestinal lipid absorption [85,86,87,88,89]. Also reported millet grains and their ethanol extract’s capacity to inhibit *de novo* FAs and cholesterol synthesis by down-regulating gene expression of SREBP-1C and its responsive lipogenic genes, including fatty acid synthase (FAS), as well as inhibiting the 3-Hydroxy-3-Methylglutaryl-CoA Reductase (HMGCR), [49]. In addition, Millets’ bioactive components can increase circulatory levels of HDL-c by inhibiting a cholesteryl ester transfer protein (CETP) [90,91,92,93].

Finally, the gut contains about 75% of our immune cells and plays a significant role in mediating systemic inflammation [94]. Currently, alterations in the gut microbiota, as well as the loss of gut integrity, are linked to a variety of intestinal and systemic inflammatory disorders, as well as the development of obesity, IR, and NAFLD [95,96,97]. A balanced diet is crucial for normal intestinal function [98]. HFD can impair intestinal mucosal membrane integrity and induce intestinal and systemic inflammation by generating ROS and activating numerous inflammatory pathways [99,100,101]. In addition, HFD-feeding mediated intestinal damage is associated with obesity, IR, and NAFLD [102,103]. The significant increase in the serum levels of LPS in the HFD-fed rats of the study is evidence of the disturbance of the gut mucosal barrier. On the other hand, it seems reasonable that MP powder and ethanolic extract could improve the intestinal integrity in HFD-fed rats and reduce levels of LPS. This supports the findings of others who have shown that the dietary fiber of millet grains could be fermented by gut microbiota and produce short-chain fatty acids (SCFAs) such as butyrate which can regulate intestinal microbiota and enhance the musical membrane integrity to reduce intestinal damage, control inflammation, and reverse fatty liver disease in its early stages [104,105,106]. However, further studies are required to confirm the intestinal protective effect of PM and to connect this protection with the obvious anti-obesity and hepatoprotective effect of this plant.

### Limitations

However, some limitations still exist in this study. Importantly, we have used a PM species that is commercially sold and cultured and cultivated during summer. Even though this was used fresh, studies have shown that the active ingredients, as well as components of the PM plant, vary with the geographic area they are grown in, environment, climate change, time of flowering, and cultivation period [107,108,109,110]. Hence, it could be much interesting to study the anti-obesity and anti-steatotic effects of different PM species collected from different parts of Saudi Arabia and other countries to confirm these effects. In addition, fractionation and HPLC studies to isolate and identify the active ingredients responsible for the observed hypoglycemic, hypolipidemic, anti-inflammatory, and hepatoprotective effects of our PM species are highly recommended and necessary in the future studies, which could reveal a novel compound that can be translated into clinical studies. Furthermore, more well-designed studies to precisely examine the molecular mechanisms behind these effects are highly recommended, such as targeting adipose and hepatic glucose and lipid regulators such as AMPK, SREBPs, and PPARs. In addition, this study highlights the importance of gut immunity in the anti-obesity, anti-inflammatory, and anti-steatotic effects of MP. Unfortunately, we were not able to investigate this further. It should be considered an excellent target for future research.

## 5. Conclusions

Our results indicated many properties of millet grains and their ethanol extract, including hepatoprotective, hypolipidemic, hypoglycemic, anti-inflammatory, and hypocholesterolemic activities in HFD-induced obesity rats. These findings may prompt nutritionists and industrial food sectors to pay closer attention to certain grains and ingrate them into their plans. Obese, overweight, and diabetic subjects may include these grains in their daily meal menus to prevent further complications and lower the risk of having CVDs.

## Figures and Tables

**Table 1:** Energy intake for both the control and HFD.

	STD (D12450H)		HFD (D12451)	
	Gm %	Kcal %	Gm %	Kcal %
Carbogydrate	67.3	70%	41	35%
Proteins	19.2	20%	24	20%
Fat	4.3	10%	24	45%
Total (Kcal/gm)	100 (3.85 Kcal/g)		100 (4.73 Kcal/g)	

## Data Availability

The datasets used and analyzed during the current study are available from the corresponding author on reasonable request.

## Supplementary Materials

The following are available online at https://www.mdpi.com/article/10.3390/nu14091791/s1, File S1: Standard curves generated by kits.
