# Supplementary material for: Anti-Hyperlipidemia, Hypoglycemic, and Hepatoprotective Impacts of Pearl Millet (Pennisetum glaucum L.) Grains and Their Ethanol Extract on Rats Fed a High-Fat Diet
Source: Nutrients. 2022 Apr 25;14(9):1791. doi: 10.3390/nu14091791 (PMC9105973; doi:10.3390/nu14091791)

Glucose

standard	Absobtion	Corrected absrobance	
0	0.045	0	
2.5	0.132	0.087	
5	0.323	0.278	
7.5	0.434	0.389	
10	0.654	0.609	
15	0.783	0.738	
20	1.065	1.02	
25	1.363	1.318	

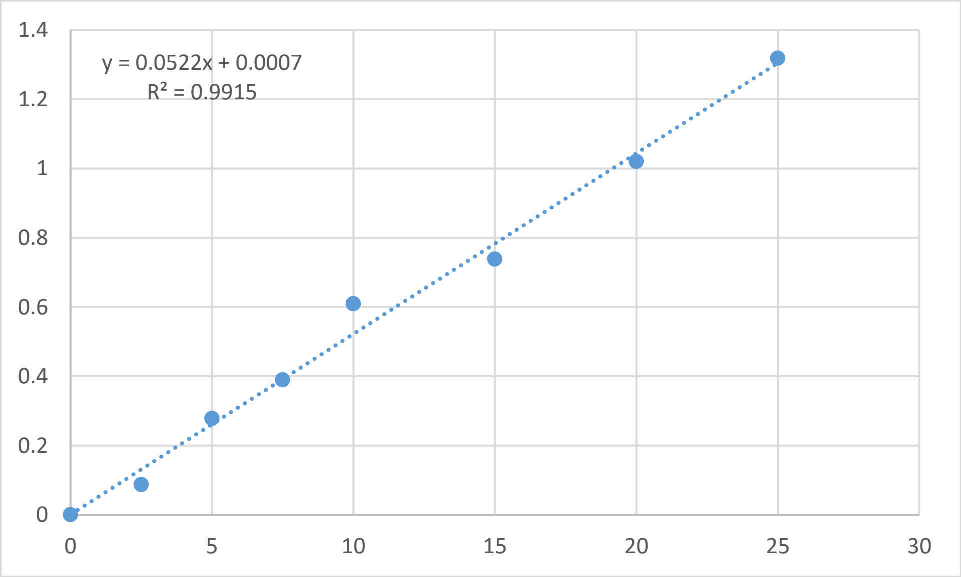

Insulin

standard	Absobtion	corrected ABS
0	0.029	0
8	0.122	0.093
16	0.332	0.303
32	0.539	0.51
80	1.381	1.352
180	2.232	2.203

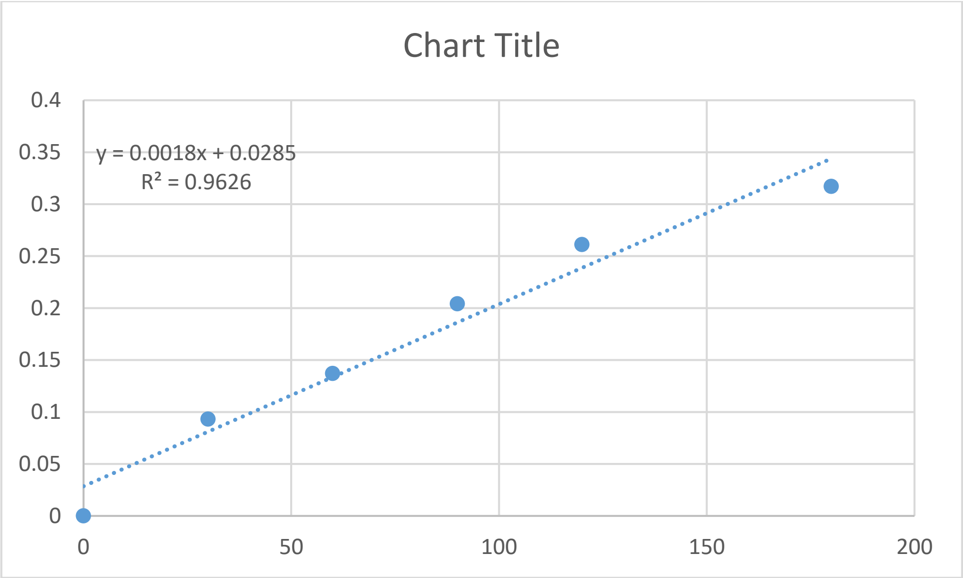

Total Cholesterol

standard (mg/dl)	Absobtion	corrected ABS
0	0.028	0
30	0.121	0.093
60	0.165	0.137
90	0.232	0.204
120	0.289	0.261
180	0.345	0.317
240	0.478	0.45
300	0.548	0.52

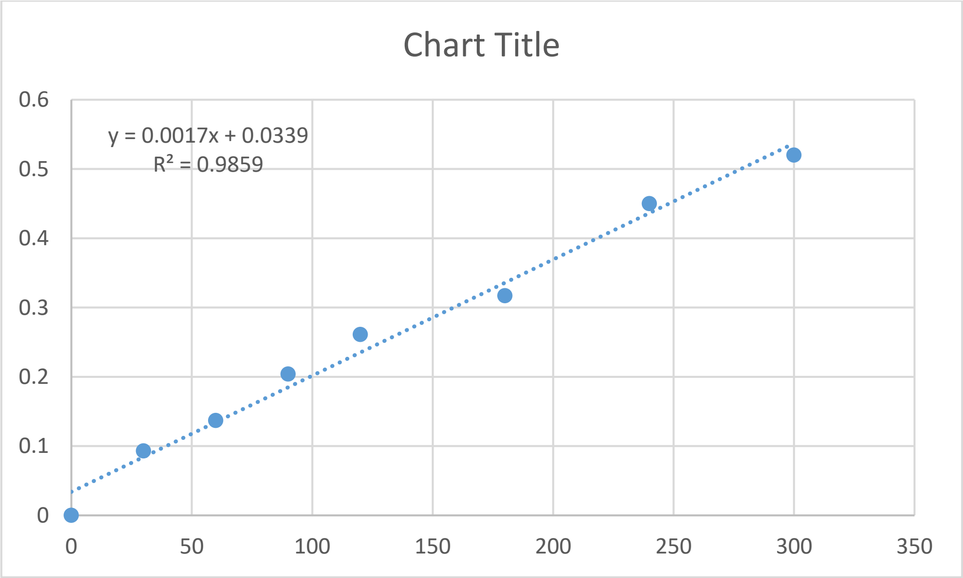

TGs

standard (mg/dl)	Absobtion	corrected ABS
0	0.016	0
3.125	0.073	0.057
6.25	0.102	0.086
12.5	0.132	0.116
25	0.198	0.182
50	0.245	0.229
100	0.389	0.373
200	0.693	0.677

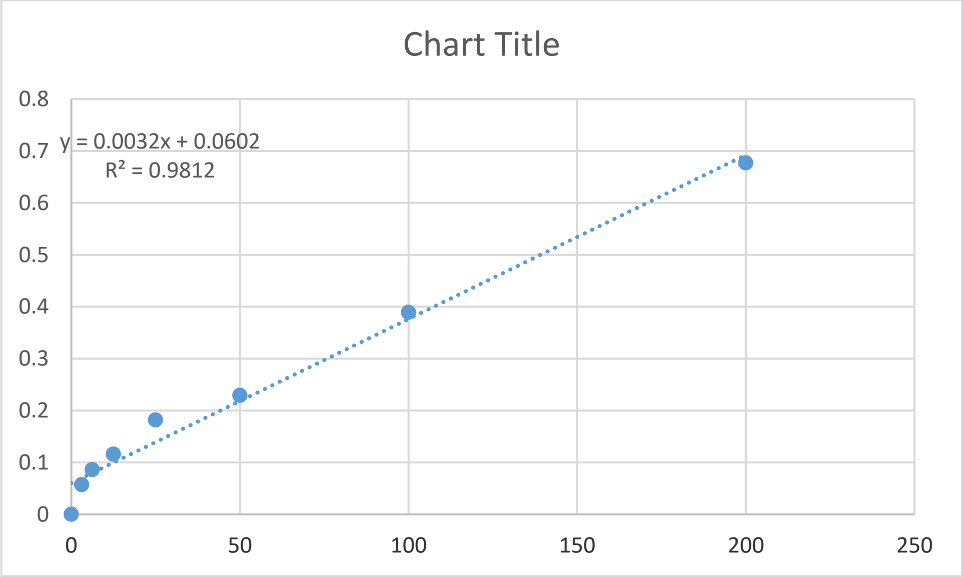

CHOL, HDL-C; LDL-c

Standard CHOL (ug/well)	Absobtion	corrected ABS
0	0.067	0
1	0.342	0.275
2	0.521	0.454
3	0.736	0.669
4	0.934	0.867
5	1.243	1.176

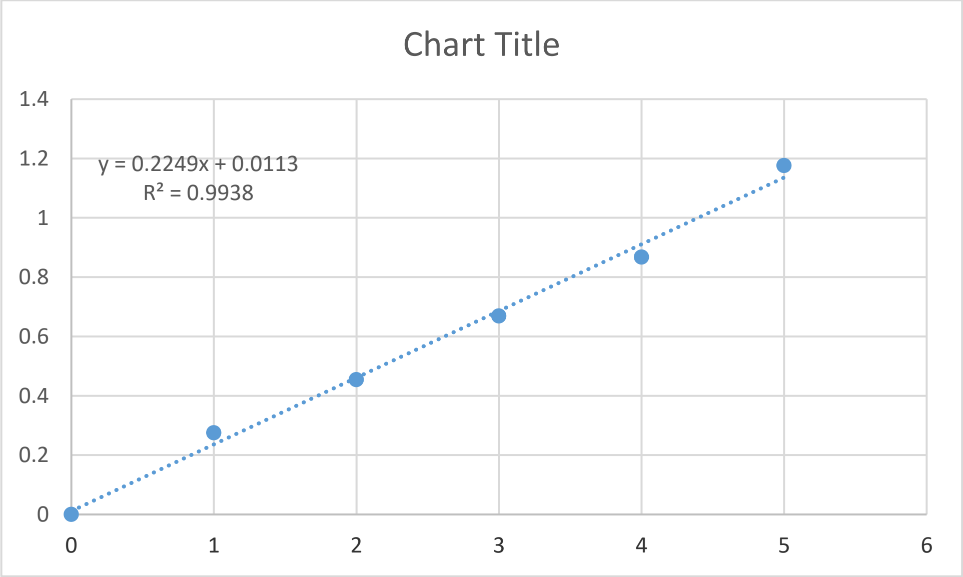

IL-6

standard (pg/ml)	ABS	ABS-background
4000	1.783	1.62
2000	1.371	1.208
1000	0.883	0.72
500	0.519	0.356
250	0.362	0.199
125	0.298	0.135
62.5	0.232	0.069
0	0.163	0

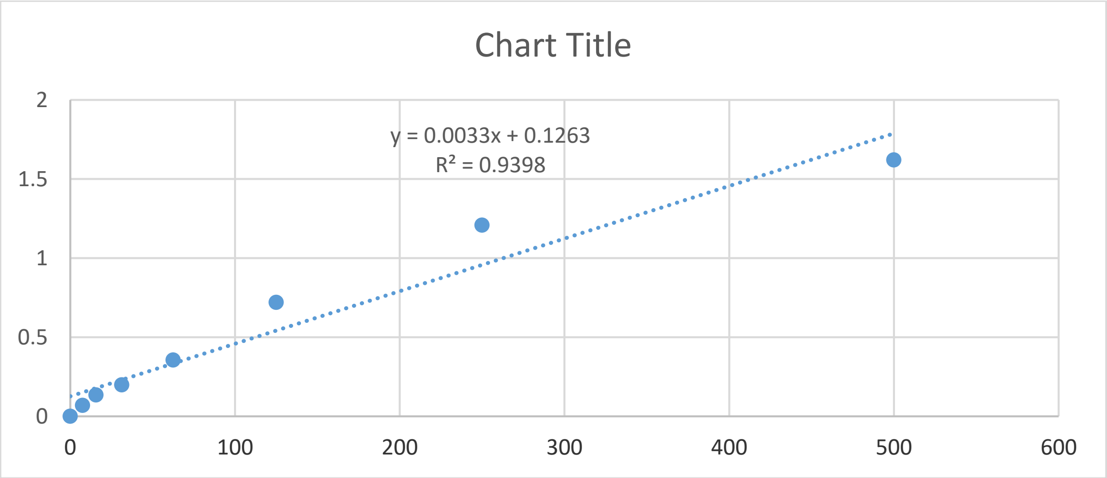

TNF-α

standard (ng/ml)	ABS	ABS-background
5000	1.878	1.8
2500	1.043	0.965
1250	0.618	0.54
625	0.468	0.39
312	0.289	0.211
156	0.176	0.098
78.3	0.082	0.004
0	0.078	0

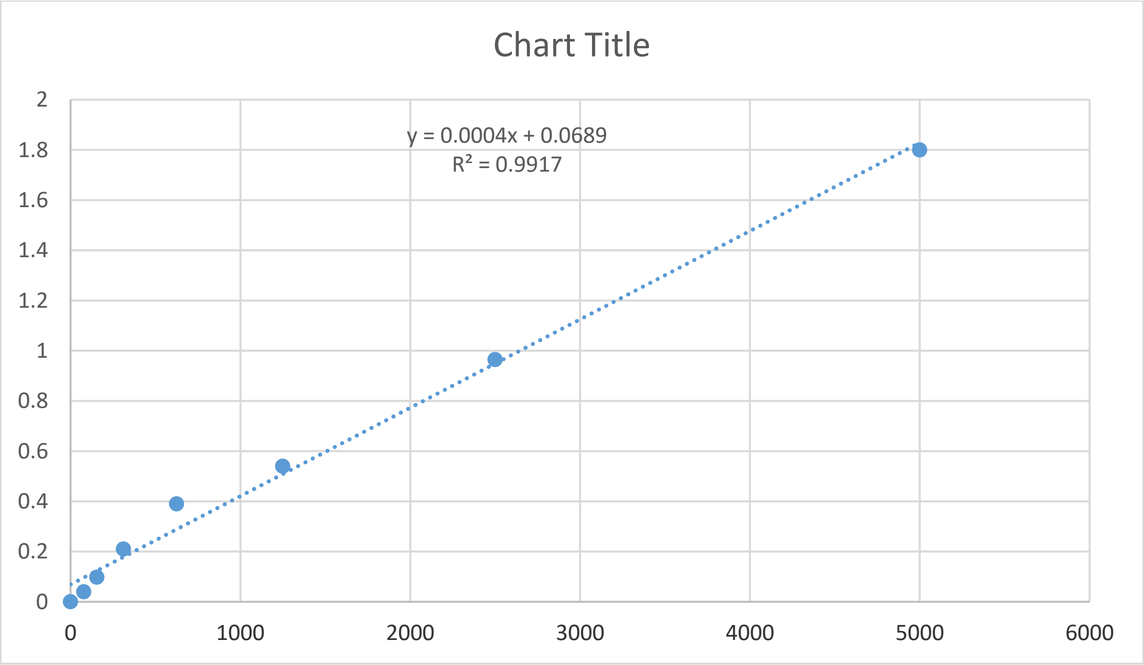

IL-10

standard (pg/ml)	ABS	ABS-background
2000	1.937	1.899
1000	1.093	1.055
500	0.798	0.76
250	0.548	0.51
125	0.303	0.265
62.5	0.198	0.16
31.25	0.122	0.084
0	0.038	0

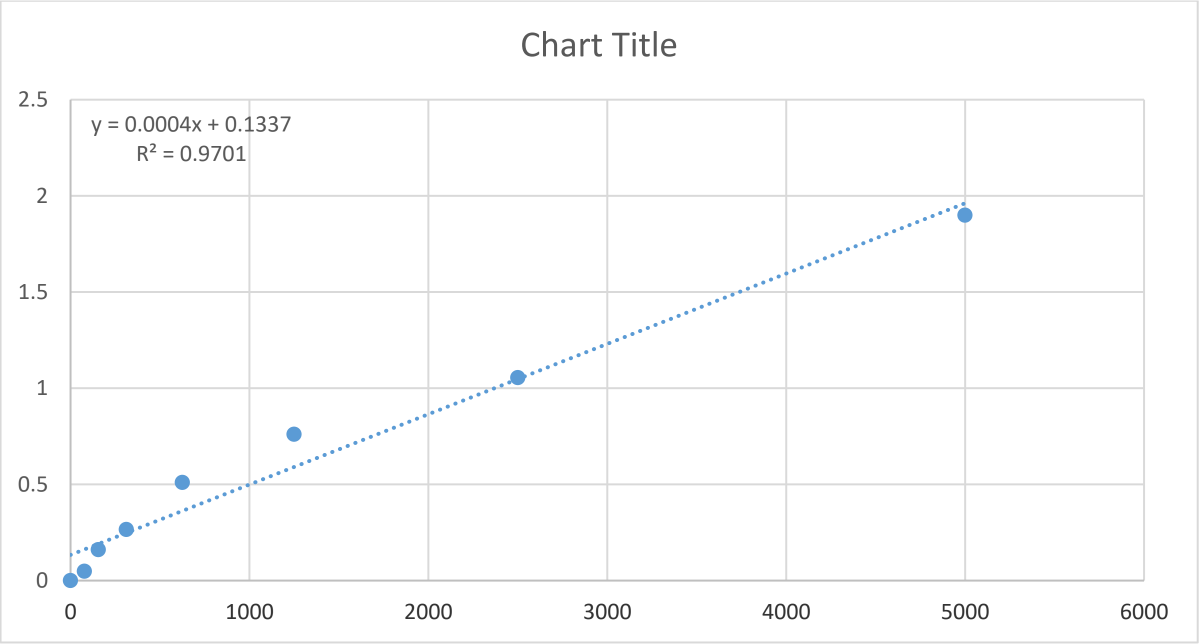

CRP

standard (pg/ml)	ABS	ABS-background
1250	3.23	3.178
625	2.32	2.268
312.25	1.093	1.041
156.3	0.763	0.711
87.1	0.383	0.331
39.1	0.253	0.201
19.5	0.124	0.072
0	0.052	0

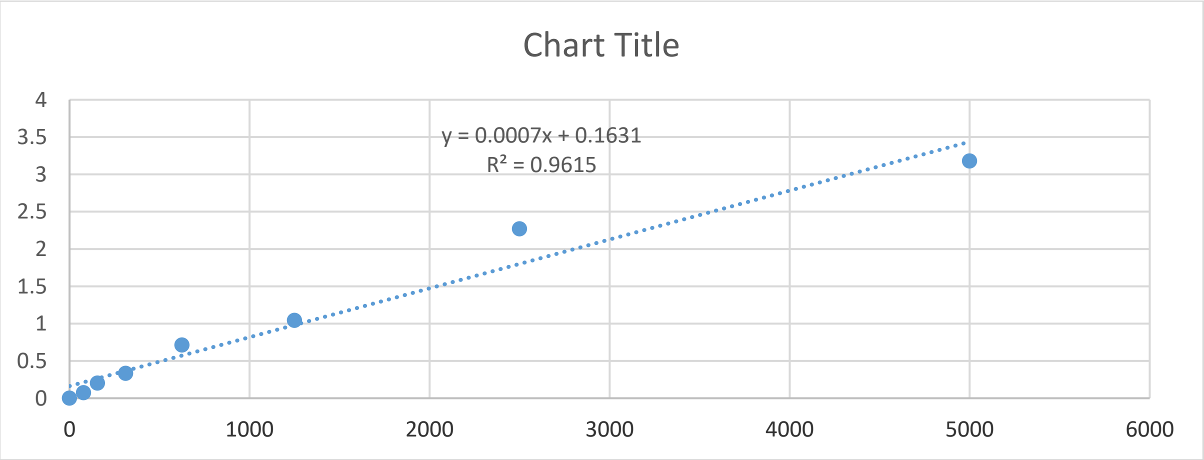

Adiponectin

standard (ng/ml)	ABS	ABS-background
600	3.114	3.082
300	1.643	1.611
150	0.829	0.797
75	0.593	0.561
37.5	0.243	0.211
18.8	0.193	0.161
9.4	0.103	0.071
0	0.032	0

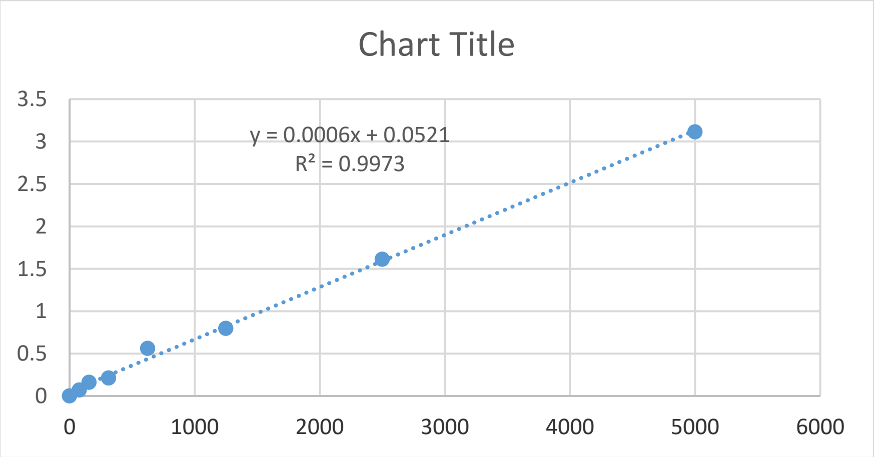

Supplement: Supplementary file 1 [file nutrients-14-01791-s001.zip › nutrients-1669733-supplementary.pdf]
